# Interferon Lambda: The Next Frontier in Antiviral Therapy?

**DOI:** 10.3390/ph18060785

**Published:** 2025-05-24

**Authors:** Sofia Chronopoulou, Ilias Tsochantaridis

**Affiliations:** Department of Molecular Biology & Genetics, Democritus University of Thrace, 68100 Alexandroupolis, Greece

**Keywords:** interferon lambda, cytokines, antiviral therapy, hepatitis B, hepatitis C, hepatitis D, COVID-19

## Abstract

Type III interferons (IFN-λ) are the most recently identified members of the interferon family, distantly related to type I interferons and members of the interleukin-10 (IL-10). Unlike type I interferons, which have broadly distributed cellular receptors, IFN-λ signals through a heterodimeric receptor complex with primary expression on epithelial cells. This restricted receptor distribution makes IFN-λ a favorable candidate for therapeutic and antiviral applications with reduced side effects. In this review, we describe the molecular structure, signaling mechanisms, and the role of IFN-λ in the innate immunity of epithelial tissue, which are its primary sites of action. Moreover, this review will summarize and critically examine the antiviral potential of IFN-λ based on all published clinical trials conducted for the treatment of COVID-19, and hepatitis B, C and D virus. Furthermore, this review suggests IFN-λ as a promising therapeutic recombinant protein, with special emphasis on its potential for production using alternative expression and advanced drug delivery systems. To emphasize its potential as a therapeutic intervention, the design and engineering of recombinant IFN-λ will be presented, with a focus on its lower side-effect profile compared to Type I interferons.

## 1. Introduction

### 1.1. Discovery and Classification of Interferon Lambda (IFN-λ)

**Figure 1:**
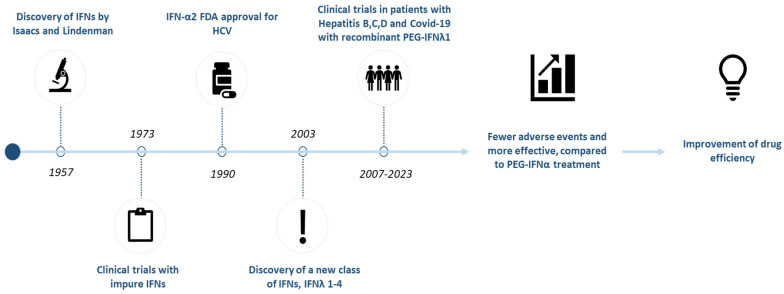
Historical overview: major milestones of interferon research and workflow of current review. IFN: Interferon; COVID-19: coronavirus disease 2019; PEG: pegylated.

Interferon lambda (IFN-λ) is among the most recently discovered members of the type III interferons, a class within the larger cytokine family known as interferons [1,2,3]. Interferons are cytokines that inhibit viral replication in host cells by initiating innate immune responses, primarily through the activation of various interferon-stimulated genes (ISGs) [4]. Based on receptor specificity, human interferons are classified into three major types: type I (IFN-α; IFN-β; IFN-δ; IFN-ε; IFN-κ; IFN-ζ; IFN-τ and IFN-ω), type II (IFN-γ) and type III (IFN-λ 1-4) [5]. A timeline of interferon development is presented in Figure 1.

When first identified in 2003, the initial three IFN-λ genes were assigned interleukin designations by the Human Genome Organization Gene Nomenclature Committee. A decade later, this classification was revised to the current gene symbols: *IFNL1* (formerly *IL29*), *IFNL2* (*IL28A*), and *IFNL3* (*IL28B*). *IFNL4*, the most recent member of the IFN-λ family, was discovered in 2013 and is expressed only in individuals carrying the DG allele of the ss469415590 variant [6]. IFN-λ genes are located on human chromosome 19 (19q13) and are highly conserved. For example, IFN-λ2 and IFN-λ3 share 96% amino acid identity, while IFN-λ1 is 81% homologous to IFN-λ2 and IFN-λ3 [7].

### 1.2. Mechanism of Action and Therapeutic Potential of IFN-λ

IFN-λ binds to a heterodimeric receptor complex consisting of IFN-λ receptor 1 (IFNLR1) and interleukin-10 receptor subunit β (IL-10Rβ), which activates the JAK-STAT signaling pathway, resulting in the phosphorylation of the signal transducer and activator of transcription proteins (STATs) and then inducing interferon-stimulated genes (ISGs) [8]. In contrast to the type I IFN receptors (IFNARs), which are expressed on various cell types, IFN-λ receptors are primarily limited to epithelial cells. IFN-λ acts as a frontline antiviral defense, being produced early and predominantly at epithelial surfaces during viral infections [9].

Type I and II interferons have been developed and clinically used for treating a number of viral infections and immune diseases (Table 1). Recombinant pegylated type I interferons, i.e., IFN alfa-2a and IFN alfa-2b, have been used to treat chronic viral hepatitis for more than 20 years. Due to the wide distribution of IFN-α receptors, these treatments lead to side effects such as flu-like symptoms, fatigue, depression, cytopenias, and autoimmune complications [10]. In contrast, type III IFN receptors are expressed in a subset of type I-responsive cells. Therefore, pharmaceutical type III IFNs are systemically related to fewer side effects, highlighting their significance in medical applications [11].

## 2. Biological Background

### 2.1. Molecular Structure, Signaling Mechanism and Common Aspects of Type III Interferons with Other Interferons

**Figure 2:**
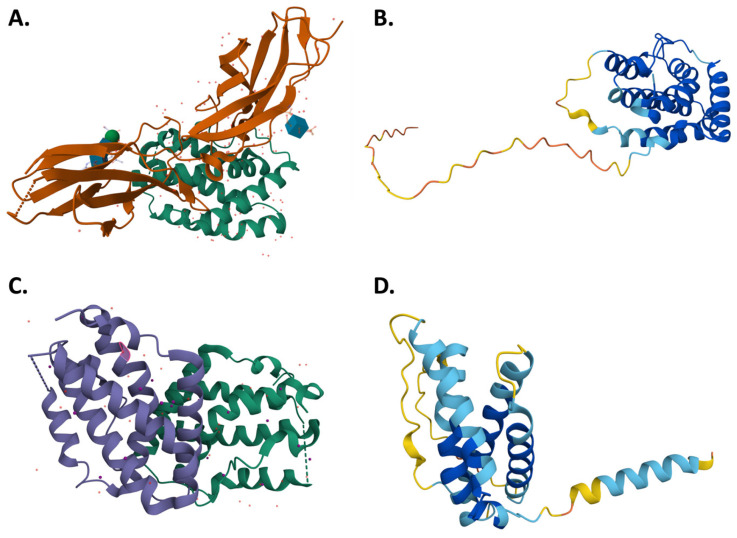
Structural representations of type III interferons. (**A**) X-ray crystallographic structure of IFN-λ1 (PDB ID: 3OG4). (**B**) Predicted structure of IFN-λ2 generated by AlphaFold (Model ID: AF-Q8IZJ0-F1). (**C**) X-ray crystallographic structure of IFN-λ3 (PDB ID: 4HHC). (**D**) Predicted structure of IFN-λ4 generated by AlphaFold (Model ID: AF-K9M1U5-F1).

At the structural level, IFN-λ is a helical cytokine with a six-helix bundle topology similar to that of the IL-10 family cytokines. The four members of the human IFN-λ family, i.e., IFN-λ1, -λ2, -λ3 and -λ4, structurally have essential motifs for the process of receptor binding. IFN-λs bind to heterodimeric receptors composed of IFNLR1 and IL-10Rβ. The α-helix-driven ligand-receptor interface determines subtype specificity and regulates the strength of downstream signaling [12]. An example schematic of structure of IFN-λ is seen in Figure 2.

**Figure 3:**
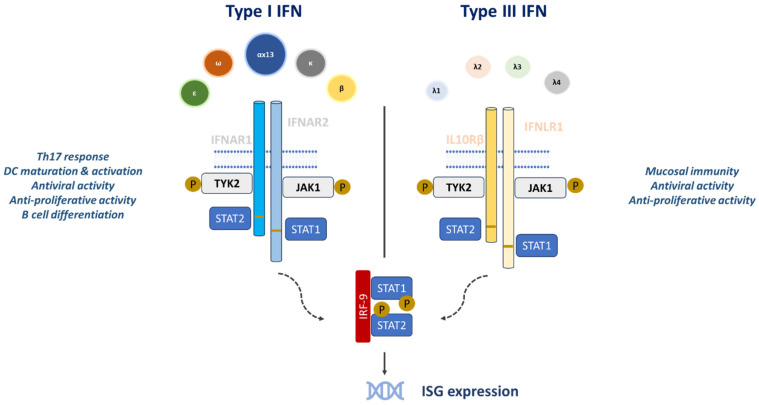
Signaling pathways induced by type I and type III Interferons. Ligand binding triggers the phosphorylation and activation of two kinases, Janus kinase 1 (JAK1) and tyrosine kinase 2 (TYK2), which in turn leads to phosphorylation of signal transducer and activator of transcription 1 (STAT1) and STAT2. Phosphorylated STAT1 and STAT2 form heterodimers and associate with interferon regulatory factor 9 (IRF9). This complex (STAT1, STAT2 and IRF9) is then imported into the nucleus triggering enhanced transcriptional activity of interferon-stimulated genes (ISGs). DC: dendritic cell.

The IFN-λ receptor (IFNLR) is a heterodimer consisting of two subunits: interleukin-10 (IL-10) receptor subunit-β (IL-10Rβ) and IFN-λ receptor 1 (IFNLR1). Ligand binding leads to phosphorylation and activation of the kinases Janus kinase 1 (JAK1) and tyrosine kinase 2 (TYK2), which in turn phosphorylate the transcription factors STAT1, STAT2 and, to a lesser extent, STAT3-STAT5 [7]. Phosphorylated STAT1 and STAT2 dimerize and, together with interferon regulatory factor 9 (IRF9), form the interferon-stimulated gene factor 3 (ISGF3) complex. ISGF3 translocates to the nucleus inducing the transcription of interferon-stimulated genes (Figure 3) [11].

Despite the structural and genetic differences between IFN-α/β and IFN-λ and their receptors, the proximal signaling processes and downstream transcriptional responses are comparable. Although the amino acid sequence of IFN-λ indicates greater similarity to IFN-α/β, its protein structure more closely resembles members of the IL-10 cytokine family. All type I IFNs signal through the heterodimeric IFNAR (IFNAR1 and IFNAR2), whereas type III IFNs bind the distinct IFNLR complex, comprising the unique subunit IFNLR1 (also termed IL-28Rα) and the shared subunit IL-10Rβ [13]. After receptor engagement, both IFN types activate JAK-family kinases, induce STAT1 and STAT2 phosphorylation, and generate ISGF3, which up-regulates various ISGs. Like IFN-α/β, IFN-λ stimulates phosphorylation of JAK1 and TYK2 but uniquely also activates JAK2, indicating additional upstream differences between the two pathways [14]. Ligand binding to IFNAR or IFNLR can further activate STAT-independent signaling cascades (MAPK and ERK pathways) and phosphorylate other STAT proteins (STAT3, STAT4, and STAT5) [15].

Type I and type III interferons induce the expression of ISGs and activate similar signaling pathways, although they exhibit different expression patterns during viral infections in vivo. Type I interferons are produced rapidly, their receptors are ubiquitously expressed and they induce ISGs more quickly and strongly than type III interferons. However, excessive type I signaling can provoke uncontrolled release of proinflammatory cytokines and chemokines, leading to immunopathological consequences. In contrast, IFNLR is expressed on a limited subset of cells and binds type III interferons with a high affinity to form a signaling-competent ternary complex [5]. Type III interferons indirectly influence T cell responses via dendritic cells due to the limited expression of IFNLR1 on T cells. Furthermore, a study has demonstrated that the addition of IFN-λ during the stimulation of peripheral blood mononuclear cells or in mixed lymphocyte reactions reduces Th2 cytokine production and enhances IFN-γ secretion [16].

### 2.2. Development and Engineering of Recombinant IFN-λ

Recombinant IFN-λ1 was initially developed at Zymogenetics (now part of Bristol-Myers Squibb). The company was the first to file patents related to IFN-λ polypeptides, expression methods as well as therapeutic applications for treating hepatitis infections. IFN-λ1 was selected as a clinical candidate based on its favorable molecular characteristics: it contains only five cysteine residues (at positions 15, 49, 112, 145 and 171), forms three disulfide bridges (C15-C112, C49-C145 and C112-C171) and has a single glycosylation site that does not appear to be necessary for biological activity [3]. In addition, human recombinant IFN-λ1 was expressed using a standard *Escherichia coli* system and demonstrated a favorable safety and pharmacokinetic profile in preclinical models, supporting its selection over IFN-λ2, IFN-λ3 and IFN-λ4. However, the *E. coli* expression system lacks eukaryotic chaperones and post-translational machinery contributing to the formation of inclusion bodies [17]. The recombinant protein includes a conservative substitution of serine for cysteine at position 171 to prevent non-native disulfide bonding, thereby improving protein folding without affecting receptor binding or biological activity. Additionally, it includes a short N-terminal deletion and the addition of an N-terminal methionine. Moreover, a 20kDa linear PEG molecule was conjugated to the N-terminal methionine of the protein to enhance its pharmacokinetic properties. These modifications improve protein stability and contribute to reduced immunogenicity. Although direct comparisons with natural IFN-λ1 are limited, PEGylation and N-terminal truncation have been suggested to mask epitopes from antibody recognition and are associated with low immunogenicity [18].

IFN-λ1 PEGylation is correlated to covalent attachment of a polyethylene glycol (PEG) molecule, which induce the reduction in renal clearance and the increase in protein half-life, thereby enhancing its pharmacokinetic profile [3]. On 20 April 2016, the clinical-stage biopharmaceutical company Eiger Biopharmaceuticals, announced the acquisition of worldwide rights to Pegylated Interferon Lambda-1 (PEG-IFN-λ1) from Bristol-Myers Squibb. Eiger planned to evaluate PEG-IFN-λ1 both as a potential monotherapy and in combination treatment for chronic hepatitis D virus (HDV) infection, the most sever and aggressive form of human viral hepatitis. Recently, the same biopharmaceutical company, under patent WO 2021/159027 A1, has investigated methods for treating SARS-CoV-2 infections using PEG-IFN-λ.

## 3. Therapeutic Applications—Ongoing Research

### 3.1. Antiviral Properties of IFN-λ and Clinical Trials

#### 3.1.1. Hepatitis B

Hepatitis B virus (HBV) is a member of the *Hepadnaviridae* family and remains a major cause of global mortality, leading to cirrhosis and hepatocellular carcinoma [19]. HBV can establish chronic infection, during which its circular DNA genome resides in the nuclei of infected hepatocytes and serves as a template for viral transcripts synthesis [20,21]. Yet, no approved therapies can completely eliminate HBV circular DNA upon the development of chronic infection status [22].

During therapy with nucleoside reverse transcriptase inhibitors (NRTIs), HBV not only persists but can also develop resistance to long-term NRTI therapy, resulting in bone and renal side-effects, This underscores the need for novel HBV therapies [23]. Since HBV circular DNA cannot be silenced by current treatments, there is increased potential for antiviral agents with novel immunomodulatory properties.

Given their key role in antiviral defense, interferons have been evaluated for their ability to suppress HBV replication. However, the majority of type I IFN-treated patients do not achieve HBV DNA suppression or seroclearance [22]. This limitation has prompted investigation into the potential of IFN-λ as a therapeutic alternative (Table 2).

In a randomized phase 2 study (LIRA-B, NCT01204762), PEG-IFN-λ was compared to pegylated interferon alfa 2a (PEG-IFNα2a) in hepatitis B e antigen (HBeAg) positive chronic HBV patients [24]. During the first 24 weeks of treatment, PEG-IFN-λ indicated faster and more substantial decrease in HBV DNA and hepatitis B surface antigen levels compared to PEG-IFNα2a, and both groups exhibited similar serologic and virologic responses by the end of the study. Nonetheless, PEG-IFNα2a demonstrated greater overall efficacy than PEG-IFN-λ (180 μg injection) based on post-treatment seroconversion, virologic suppression and biochemical response rates [24]. During this trial, a separate group of 13 patients received entecavir (an NRTI) for 12 weeks prior to PEG-IFN-λ treatment. This small-scale analysis indicated that in certain patients with reductions in HBV DNA and HBeAg, treatment with IFN-λ was associated with enhanced immunostimulatory effects. Specifically, it promoted natural killer cell polyfunctionality and maintained HBV-specific CD4+ and CD8+ T cells [25]. These findings suggest a potential role for IFN-λ, especially when combined with NRTIs, in supporting immune-mediated control of HBV, which may lead to the suppression of cccDNA activity.

Nonetheless, further clinical trials must be conducted to unravel the immunomodulatory mechanisms of IFN-λ and clarify its impact on HBV DNA regulation.

#### 3.1.2. Hepatitis C

Hepatitis C virus (HCV) is a small, single-stranded RNA virus belonging to *Flaviviridae* family [26]. It was discovered in 1988 by Michael Houghton’s team [27], and over the past 30 years, significant progress has been made in the development of serological and virological diagnostic tests as well as therapeutic approaches. Based on phylogenetic and sequence analyses, HCV has been classified into 7 major genotypes, among which genotypes 1, 2 and 3 are the most widespread and extensively studied [28].

The rapid development of diagnostic tools and therapeutic approaches is driven by the epidemiological burden of viral hepatitis. Viral hepatitis is one of the main leading causes of death worldwide, in comparison with HIV infection, tuberculosis and malaria [29]. The primary aim of antiviral therapy for chronic HCV infection is viral suppression, which is defined by the absence of HCV RNA in serum 12–24 weeks after the treatment completion [19].

Standard interferon alfa (IFN-α) treatment achieved sustained virologic response (SVR) rates of only 5–20%. Therefore, IFN-α was approved in the United States in 1992 as a treatment for chronic HCV infection [30]. In 1998, the nucleoside analog ribavirin (RBV) was approved in combination with IFN-α for the treatment of chronic HCV, leading to improved SVR rates more than twofold compared to IFN-α alone [31]. In 2001, pegylated IFN-α was approved for the treatment of chronic HBV and HCV. Pegylation of IFN-α (PEG-IFNα) resulted in higher and longer-lasting serum concentrations while the addition of ribavirin resulted in even higher SVR rates than with unpegylated IFN-α [32,33]. However, the side effects and biological responses associated with IFN-α prompted interest in IFN-λ as an alternative therapeutic agent.

In vitro toxicology studies by ZymoGenetics, strongly suggested that PEG-IFN-λ is less likely to induce the hematologic toxicities commonly related to PEG-IFNα [34]. Based on preclinical studies, PEG-IFN-λ was evaluated in clinical studies as a potential therapeutic agent for chronic HCV infection. The first human trial (phase 1a) was a randomized, blinded, placebo-controlled study, in which 24 healthy volunteers received a single subcutaneous dose of PEG-IFN-λ at different concentrations (0.5, 1.5, 5.0 or 7.5 μg/kg) or placebo. Overall, PEG-IFN-λ was well-tolerated, except at doses up to 5.0 μg/kg, with minimal adverse effects (fatigue, hematologic changes). Only a few participants developed dose-dependent increase in aminotransferase levels [34].

Still, little is known about the specific patient characteristics that may predict adverse outcomes or differential responses to PEG-IFN-λ treatment. Safety and efficacy profiles could potentially be affected by different factors such as baseline liver function, presence of comorbidities and immune status [35]. Future clinical studies with larger and more diverse patient populations are essential to identify predictors and optimize patient selection for PEG-IFN-λ therapy.

A subsequent phase 1b trial in patients with HCV genotype 1 evaluated the safety and antiviral efficacy of repeated doses of PEG-IFN-λ with or without ribavirin. Over 4 weeks of treatment, the study revealed significant dose-related reductions in HCV RNA levels. When combined with ribavirin, even greater reductions in viral levels were observed [36].

A phase 2a trial assessed fixed doses of PEG-IFN-λ (80, 120, 180 or 240 μg) plus ribavirin, in 55 HCV patients (genotypes 1–4) and compared the outcomes of PEG-IFNα2a (180 μg) plus ribavirin [3]. At weeks 4 and 12, the proportion of patients with undetectable HCV RNA levels was comparable between PEG-IFN-λ and PEG-IFNα2a groups. Moreover, this trial confirmed that PEG-IFN-λ had an effective dosing range (120–240 μg) and emphasized the utility of fixed-dose regimens [3].

These findings led to a larger, randomized, blinded, phase 2b trial (EMERGE; NCT01001754) [37], enrolling 525 treatment-naïve patients with HCV genotypes 1–4. More specifically, patients received weekly subcutaneous single doses of 120, 180 or 240 μg of PEG-IFN-λ or 180 μg PEG-IFNα2a all in combination with ribavirin. Results revealed that PEG-IFN-λ/ribavirin (180 μg) for 24 or 48 weeks resulted in SVR rates similar to PEG-IFNα2a/ribavirin, with improved early virologic response. and fewer systemic and hematologic side effects (Table 2) [37].

Furthermore, the NCT01616524 phase 3 trial was a randomized, controlled study comparing PEG-IFN-λ plus ribavirin, with or without daclatasvir (DCV, a direct-acting antiviral and NS5A replication complex inhibitor) to PEG-IFNα2a plus ribavirin. More precisely, 874 patients with HCV genotypes 2 and 3 were enrolled to receive one of the following regimens: (1) 24 weeks of 180 μg PEG-IFN-λ plus 400 mg RBV and a DCV placebo for 12 first weeks followed by 60 mg DCV once daily; (2) 12 weeks of 180 μg PEG-IFN-λ plus 400 mg RBV and 60 mg DCV once daily; or (3) 24 weeks of 180 μg PEG-IFNα2a plus 400 mg RBV and a DCV placebo for 12 first weeks [38]. Both PEG-IFNs were administered subcutaneously at a dose of 180 μg once weekly. The efficiency and safety of PEG-IFN-λ combined with ribavirin and daclatasvir were consistent with findings from previous studies (2D-LITE trial, NCT01309932, Muir et al. 2014) [37]. This large, multinational phase 3 trial demonstrated that 12 weeks of treatment with PEG-IFN-λ plus RBV and DCV resulted in a superior SVR12 rate compared to 24 weeks treatment of PEG-IFNα2a plus RBV [38].

Another randomized, double blind phase 3 trial (NCT01598090) evaluated the efficacy and safety of PEG-IFN-λ/RBV in combination with another direct-acting antiviral, telaprevir, in patients with genotype 1 chronic HCV infection, either treatment naïve or relapsed following prior PEG-IFNα/RBV treatment. Patients received either 180 μg of PEG-IFN-λ or PEG-IFN-α plus RBV and telaprevir for 24 or 48 weeks. At the end of this study, the results showed that PEG-IFN-λ was less effective than PEG-IFNα in achieving key treatment outcomes, including SVR and biochemical remission [39].

The efficacy and safety of PEG-IFN-λ/RBV/DCV was further assessed in a multinational phase 3 trial (NCT01866930) in patients with chronic HCV (genotypes 1–4) coinfected with human immunodeficiency virus (HIV). Treatment- naïve patients received subcutaneously 180 μg PEG-IFN-λ once weekly for 12 weeks as well as weight-based RBV and DCV (in proportions based on the concomitant HIV regimen). Post-treatment strategies varied by HCV genotype: patients with genotypes 2 or 3 received an additional 12 weeks of PEG-IFN-λ/RBV therapy with 24 weeks of follow-up, while patients with HCV genotypes 1 (a or b) or 4 received response-guided therapy. At the end of this study, SVR rates ranged from 72% to 95%, depending on the genotype of individuals and the regimen was generally well tolerated. Only a few participants discontinued due to adverse effects [40].

Another similar, phase 3 trial (NCT01741545) evaluated the efficiency of PEG-IFN-λ/RBV/DCV in hemophiliac patients with chronic HCV, a population traditionally considered difficult to treat due to various poor prognostic factors. Treatment-naïve individuals enrolled in this study were classified in 2 groups based on HCV genotypes (genotype 2 or 3 was cohort A while genotype 1b or 4 was cohort B). Both groups received 180 μg of PEG-IFN-λ subcutaneously once weekly, weight-based RBV and 60 mg DCV daily for 12 weeks. Patients in cohort B received an additional 12 weeks of PEG-IFN-λ/RBV treatment. This phase 3 trial demonstrated higher SVR12 rates in both cohorts, compared to those typically observed in PEG-IFN-α/RBV therapy [41].

Despite the encouraging results from the clinical trials mentioned above, further development of PEG-IFN-λ for HCV treatment was discontinued due to the emergence of highly effective oral direct-acting antiviral combinations, which now achieve SVR rates of up to 99% [42].

#### 3.1.3. Hepatitis D

Discovered in 1977 by Rizzetto and colleagues [43], hepatitis delta virus (HDV) is the smallest known human-infecting virus [44]. With an RNA genome of approximately 1700 nucleotides [44], HDV requires prior HBV infection to propagate, as it lacks the ability to encode its own envelope proteins and depends on the expression of HBV surface antigen in the same host cell [44].

Chronic hepatitis D (CHD), caused by HDB, is considered the most severe and aggressive type of viral hepatitis [45]. Currently, HDV treatment relies on IFN-α, which has limited efficacy and is often related to post-treatment relapses. Additionally, nucleos(t)ide analogs, originally developed for HBV therapy, have no effect on HDV replication [46]. However, several novel therapeutic approaches are under investigation. These include agents targeting viral assembly and release (lonafarnib and REP2139), viral entry (Myrcludex B) and immunomodulation through indirect activation of the innate immune system, such as with PEG-IFN-λ [47]. PEG-IFN-λ is currently being evaluated in clinical trials both as monotherapy (NCT02765802) and in combination with other agents (NCT03600714) (Table 2).

In a phase 2 open-label study (LIMT-1, clinical trial NCT02765802), 33 patients were randomized to receive subcutaneous PEG-IFN-λ at doses of either 120 μg or 180 μg weekly for 48 weeks, followed by a 24-week observation period. In patients receiving the 180 μg dose, serum HDV RNA levels declined by 2.4 log, a reduction comparable to the ~2.5 log decline historically observed with PEG-IFNα. Interestingly, a dose-dependent 1.4 log reduction in serum HDV RNA levels was observed in patients receiving 120 μg of PEG-IFN-λ [48]. The side effects, such as flu-like symptoms and hyperbilirubinemia, were less frequent and less severe in comparison with the side effects of PEG-IFNα treatment [49]. The LIMT-2 phase 3 clinical trial (NCT05070364) was initiated to evaluate the safety and therapeutic potential of PEG-IFN-λ administered at 180 μg per week over a 48-week period. Despite initial progress, the trial was halted on 7 September 2023, after the Data Safety Monitoring Board reported four instances of hepatobiliary adverse events resulting in liver decompensation.

In another phase 2 trial, the combined treatment of PEG-IFN-λ and lonafarnib was used to enhance antiviral responses. More specifically, the LIFT-1 study (NCT03600714) comprised 26 CHD patients who received 50 mg lonafarnib orally and 100 mg ritonavir twice daily in combination with 180 μg PEG-IFN-λ subcutaneously once weekly for 24 weeks, followed by a 24-week post-treatment period. At the end of the treatment, 50% of patients had serum HDV RNA levels below the lower limit of quantification. Nevertheless, 23% of patients experienced virologic relapse [50]. A follow-up phase 2 LIFT-2 trial will be conducted to assess the long-term efficacy of this combination with an extended 48-week treatment duration.

CHD remains the most severe form of human viral hepatitis. CHD is more severe and aggressive than other viral hepatitis types primarily because HDV requires HBV for replication, leading to coinfection that accelerates liver damage. The immune response against HDV is typically more robust and inflammatory, resulting in faster fibrosis progression, earlier cirrhosis onset, and increased hepatocellular carcinoma risk [51]. Although PEG-IFN-λ could be a promising novel therapeutic option, further studies need to be performed to explore its efficiency as monotherapy or in combination with other agents.

#### 3.1.4. Hepatitis E

Hepatitis E virus (HEV), a member of the *Hepeviridae* family, is a small, non-enveloped single-stranded RNA virus [52]. HEV is transmitted enterically resulting in up to 70,000 deaths worldwide each year. There are eight identified genotypes (gt) of HEV, of which gt 1–4 and gt7 are known to infect humans [53]. Chronic infection with HEV gt3 has emerged as a significant health problem in transplant recipients receiving immunosuppressive therapy [54,55].

Although antiviral agents such as ribavirin or PEG-IFNα are currently used for treatment, both are associated with severe side effects and there is still no optimal treatment for chronic HEV infection.

Given that PEG-IFN-λ has been clinically tested as an antiviral agent for chronic hepatitis infections, including hepatitis B, C and D infections, it has also been proposed as a potential the treatment for HEV. In pre-clinical studies using a humanized liver mouse model, PEG-IFN-λ demonstrated for the first time in vivo antiviral activity against persistent HEV gt3 infection [56].

Notably, no side effects were observed after treatment for >8 weeks and for up to 0.3 mg/kg of PEG-IFN-λ [56]. However, further investigation is needed to determine whether these findings can be applied to patients.

#### 3.1.5. COVID-19

First reported in Wuhan (China), at the end of 2019, coronavirus disease (COVID-19), caused by Severe Acute Respiratory Syndrome Coronavirus 2 (SARS-CoV-2), has led to more than 7 million deaths, as of April 2024 (https://www.worldometers.info/coronavirus/coronavirus-death-toll/ (accessed on 24 April 2024)). Although widespread vaccination has significantly reduced COVID-19 cases, the emergence of new variants and the rapid transmission of the virus highlight the continued need for effective therapeutics to reduce viral shedding, alleviate symptoms and prevent hospitalization. Although the administration of antiviral drugs such as ritonavir, nirmatrelvir, remdesivir, and molnupiravir as well as monoclonal antibodies against the spike protein of SARS-CoV-2 (bebtelovimab) are currently recommended by the US Centers for Disease Control and Prevention, these treatments have severe limitations. Monoclonal antibodies, may lose efficacy due to viral mutations, while antivirals are often difficult to administer and not widely available [57].

One of the major challenges in managing SARS-CoV-2 infections is the continuous evolution of the monoclonal antibody- and vaccine-targeted viruses, especially in the spike (S) glycoprotein. The spike-protein-based mutations could lead to alteration in protein structure, stability, neutralizing antibody interactions, receptor binding domain flexibility and accessibility to the human ACE2. Consequently, the mutation profile of the spike protein complicates the use of antibody-based interventions, prompting investigations into potentially alternative therapies such as IFNs. A recent meta-analysis has assessed the impact of these mutations on viral infectivity and immune evasion mechanisms [58].

IFNs, as key components of the initial defense against viral infections, have emerged as promising therapeutic candidates against SARS-CoV-2. In vitro studies revealed that both type I and III IFNs inhibit viral replication, providing a strong rationale for administration in humans [59]. To reduce the adverse effects commonly associated with type I IFNs, clinical trials have investigated PEG-IFN-λ as an alternative therapeutic option. Two randomized placebo-controlled trials (COVID-LAMDA and ILIAD trial) generated contradictory results (Table 3). The COVID-LAMBDA trial (NCT04331899) reported that a single subcutaneous dose of 180 μg PEG-IFN-λ did not significantly reduce the duration of SARS-CoV-2 viral shedding or improve clinical symptoms [60]. On the contrary, ILIAD trial (NCT04354259) showed that a single subcutaneous injection of 180 μg PEG-IFN-λ accelerated viral clearance as determined by RT-PCR in nasopharyngeal swabs [61]. The contradictory results of these trials may be attributed to differences in baseline viral load, timing of administration and patient demographics. Subsequent studies have demonstrated that PEG-IFN-λ is more effective in individuals with high viral load and when administered early in the course of injection [60,61].

Recently, a phase 3 clinical trial (NCT04727424) reported that a single dose of PEG-IFN-λ administered to predominantly vaccinated adults reduced the incidence of hospitalization due to COVID-19, likely through both accelerated viral clearance and favorable modulation of the innate immune response at epithelial surfaces [62]. Another clinical trial in hospitalized patients with mild COVID-19 (NCT04343976) indicated that a double dose of PEG-IFN-λ may enhance viral load decline [57]. Despite the promising results of multiple clinical trials, PEG-IFN-λ has not yet received regulatory approval for the treatment of prevention of COVID-19 in any country.

Beyond therapeutic use, the prophylactic potential of PEG-IFN-λ is also being explored. The PROTECT trial (NCT04344600) is currently evaluating whether a single subcutaneous dose can prevent SARS-CoV-2 infection in high-risk non-hospitalized individuals with household exposure. Given its mechanism of enhancing localized antiviral immune responses with limited systemic side effects, PEG-IFN-λ has been proposed as a complementary agent to direct-acting antivirals and vaccines. Such combination strategies are currently under clinical investigation to maximize therapeutic efficacy and broaden protection [63].

In conclusion, the majority of clinical trials indicate that treatment with PEG-IFN-λ accelerates SARS-CoV-2 viral clearance, especially in patients with high baseline viral load. At present, PEG-IFN-λ is among the most promising antiviral therapies under investigation against COVID-19. Ongoing trials, including NCT04534673 and Phase-2b of NCT04354259, aim to further assess the clinical efficacy of single or double subcutaneous dose of PEG-IFN-λ in hospitalized patients with moderate disease.

### 3.2. Other Potential Applications

#### 3.2.1. Cancer

In addition to their antiviral activity, interferons have been extensively studied and for their antitumor efficacy. IFN-α has demonstrated antiproliferative, pro-apoptotic and immunomodulatory properties in many cancer models [64] and has also been used clinically to treat malignancies such as melanoma [65]. However, high doses of IFN-α, with significant side effects, need to be administered to patients for achieving these therapeutic benefits [66].

To overcome the toxicity associated with IFN-α treatment, the antitumor potential of IFN-λ has been investigated. Independent studies have confirmed the antitumor activity of IFN-λ in melanoma and in other tumor models including lung [67], hepatoma [68], esophagus [69], breast [70] and prostate [71] cancers as well as colon adenocarcinoma [72] and MCA205 fibrosarcoma cell tumor models [73].

A comparative study in a mouse hepatoma model revealed that the combination of IFN-α and IFN-λ significantly improved therapeutic outcomes. Unlike IFN-α monotherapy, IFN-α/λ combination therapy resulted in complete tumor regression [74]. Although IFN-λ may be considered an alternative treatment in cases where tumors are resistant to IFN-α, it might be more beneficial to be administered in the clinic in combination with low-dose IFN-α rather than as a monotherapy. Further investigation on the interaction between IFN-α and IFN-λ within the tumor microenvironment, in combination with clinical trials evaluating their combined administration, may open new avenues in cancer therapy.

#### 3.2.2. Autoimmune Diseases

Despite their important role in suppressing viral replication, IFNs have also been implicated in autoinflammatory diseases [75]. Elevated levels of IFN-α and IFN-λ have been found in the blood and affected tissues of patients with autoimmune rheumatic diseases, such as systemic lupus erythematosus (SLE) [76,77] and rheumatoid arthritis [78], suggesting that chronic and excessive expression of these interferons may contribute to disease pathology.

Recent data revealed that recombinant IFN-λ can suppress inflammation by modulating neutrophil function in mouse models of arthritis, colitis and thrombo-inflammation [79,80]. Still, it remains unclear if IFN-λ has similar effects on human neutrophils or whether its clinical use is associated with adverse events. Growing research suggests that type III interferons cannot be strictly categorized as proinflammatory or anti-inflammatory. On the contrary, their effects seem to be context-dependent, varying by disease environment. This underscores the need for further studies to optimize the therapeutic value of IFN-λ in autoimmune rheumatic diseases [81,82]. Further clinical trials with PEG-IFN-λ will be essential to determine its efficacy and safety, and to guide its application in the management of autoimmune manifestations.

#### 3.2.3. Bacterial Infections

Recent studies have indicated that IFN-λ is implicated in different bacterial infections such as *P. aeruginosa*, *S. aureus*, and *K. pneumoniae* [83]. In adult IFNLR1-defecient mice, the absence of IFN-λ signaling was correlated with reduced infection-related pulmonary pathology and increased bacterial clearance [84,85,86]. Moreover, IFN-λ caused severe lung damage in *B. pertussis*-infected mice, with no impact on bacterial burden [87]. In the case of *S. aureus* infection, IFNLR1-deficient mice had reduced IL-1β release by neutrophils as well as decreased bacterial loads and lung pathology in comparison with infected wild-type mice [84]. In *K. pneumoniae* infection, IFN-λ triggered significant disruptions in the epithelial barrier integrity, thus facilitating the recruitment of immune cells and promoting bacterial dissemination from the respiratory tract [86]. These findings suggest that IFN-λ effects in bacterial infections are context-dependent and further studies are required to explore its precise therapeutic potential.

## 4. Future Perspectives and Innovations

To enhance the therapeutic potential of IFN-λ and strengthen its signaling efficacy, Mendosa et al. developed H11, a high-affinity variant of type III IFN, using yeast display, a widely used method in cytokine engineering [12]. Nevertheless, type III IFN signaling remains less potent than that of type I IFNs. In the same context, Yu and colleagues developed 2 analogs by swapping fragments of IFNλ1 and IFNλ3. These variants exhibited improved antiviral activity and stability, although their effects on the 3D structure and consequently on ligand-receptor affinity remain unclear [88].

Additionally, efforts to enhance the efficacy of IFN-λ using recombinant DNA technology may involve combining optimized expression systems with advanced drug delivery strategies. One promising expression system is the eukaryotic yeast *Pichia pastoris*, a methylotrophic yeast known for its low-cost, high yield production of recombinant proteins with high similarity of glycosylation to mammalian cells [89]. *P. pastoris* is preferred over the simple hosts such as *E. coli* and *S. cerevisiae* for generating complex recombinant proteins mainly due to its ability to produce compounds with mammalian glycosylation profiles [90]. Several industries around the world have expressed interest in utilizing *P. pastoris* over *E. coli* for heterologous production of recombinant proteins because it has been shown to be cost effective, simple and fast in delivering high protein expression levels while also achieving the maximal success rate in terms of recombinant protein expression. It is very simple to manipulate, similar to *E. coli* and it is inexpensive. It also has a high efficiency rate for protein expression. *P. pastoris* expression system is less expensive than E.coli and exhibits similarities with CHO cell lines of eukaryotic expression systems [91]. A recent study conducted by our research team successfully expressed the human cytokine IFN-α2a in *Pichia pastoris* and confirmed its biological activity [92]. The formation of inclusion bodies (IB), protein-based aggregates naturally formed because of cell stress related to the overproduction of heterologous proteins, is common not only to *E. coli* but also in *P. pastoris* [93]. There are several strategies to avoid or minimize IB formation, including the optimization of expression conditions, the utilization of solubility-enhancing tags, the secretion of a heterologous protein in the culture medium, the co-expression of chaperones during the production process and the use of mutant strains [93].

Interestingly, *P. pastoris*-generated IFN-α2a exhibited increased biological activity compared to FDA-approved IFN-α2a, expressed in *E. coli*. Applying a similar strategy to produce human IFN-λ could have the same benefits, especially when coupled with advanced drug delivery systems, such as hydrogels [94]. Hydrogels are comprised of natural, semi-synthetic or synthetic polymers that are physically or chemically crosslinked to form a highly hydrated mesh network [95]. These systems provide several advantages, including drug protection, biocompatibility and spatiotemporal control over drug release [95]. Moreover, they are adaptable with various administration routes, including topical/transdermal, ocular, oral, and local parenteral delivery [96,97,98,99]. In case of IFN-λ, the versatility of hydrogel-based drug delivery systems is particularly relevant, considering that, in the majority of clinical trials, a single dose is administered once weekly. This delivery strategy prevents burst drug release, ensures sustained therapeutic levels, minimizes side effects and expands the therapeutic potential of IFN-based treatments.

## 5. Conclusions

Identified approximately 22 years ago, type III IFNs have emerged as highly promising cytokines for drug development. Although they signal through the same pathway as type I IFNs, IFN-λs avoid many of the type I IFN-associated side effects, due to the restricted expression of type I IFN receptors. Since their discovery, numerous clinical trials have been conducted using PEG-IFN-λ1 in patients with viral infections.

Recent clinical studies summarized in this review highlight the significant protective role of IFN-λ in antiviral defense and its advantages compared to the type I IFNs, in terms of reduced side effects and enhanced efficacy. In the case of hepatitis C, the introduction of DAAs has revolutionized the treatment of chronic infection, leading to the suspension of PEG-IFN-λ development. In contrast, hepatitis B is a more complex therapeutic challenge that requires further investigation. However, at present, PEG-IFN-λ is considered one of the most promising antiviral therapies under evaluation against COVID-19 and hepatitis D.

Beyond hepatitis and COVID-19, IFN-λ plays a crucial role in modulating disease outcomes across a broad spectrum of infections. For example, studies in murine models revealed that IFN-λ had a decisive role in the immunological protection against norovirus [100], West Nile virus [13,101], Zika virus [102] and influenza virus [103]. Interestingly, emerging evidence also suggests a role for IFN-λ in defense against bacterial and fungal infections [104], as well as in potential applications for cancer and autoimmune diseases. Hence, IFNλ1 shows exciting therapeutic potential.

## Figures and Tables

**Table 1:** List of currently available therapeutic interferons.

Interferon Type	Drug Brand Name	Manufacturing Company	Market Entry Date	Disease
IFNα-2a	Pegasys	Pharmaand GmbH	16 October 2002	Chronic hepatitis B and C
IFNα-2b	Besremi	PharmaEssentia USA Corporation	12 November 2021	Polycythemia vera
IFNα-n3	Alferon N	AIM ImmunoTech Inc.	10 October 1989	Condyloma acuminate
IFNβ-1a	Avonex	Biogen Inc.	23 May 2003	Multiple sclerosis
	Rebif	EMD Serono, Inc.	7 March 2002	Multiple sclerosis
	Plegridy	Biogen Inc.	15 August 2014	Multiple sclerosis
IFNβ-1b	Betaseron	Bayer HealthCare Pharmaceuticals Inc.	11 August 2009	Multiple sclerosis
	Betaferon	BayerPharma	30 November 1995	Multiple sclerosis
IFNγ-1b	Actimmune	Horizon Therapeutics Ireland DAC Dublin, Ireland	1 December 2013	Malignant osteopetrosis; Chronic granulomatous disease

Data from National Library of Medicine (https://www.nlm.nih.gov, accessed on 29 November 2024).

**Table 2:** Overview of clinical trials of pegylated-interferon lambda in patients infected with hepatitis B, C, and D virus.

Condition	Clinical Trial Number	Trial Phase	Number of Participants Treated	Intervention	Publication Date
HDV infection	NCT02765802	Phase 2	33	120 μg or 180 μg PEG-IFNλ	2023
HDV infection	NCT03600714	Phase 2	26	180 μg PEG-IFNλ + LNF + RTV	2020
HBV infection	NCT01204762	Phase 2	163	180 μg PEG-IFNλ or 180 μg PEG-IFNα2a	2015
HCV (GT 1-4) + HIV infection	NCT01866930	Phase 3	300	180 μg PEG-IFNλ + RBV + DCV	2016
HCV infection GT 2 or 3	NCT01616524	Phase 3	874	180 μg PEG-IFNλ + RBV/RBV + DCV or 180 μg PEG-IFNα2a + RBV	2016
HCV infection GT 1	NCT01598090	Phase 3	617	180 μg PEG-IFNλ + RBV + TVR or 180 μg PEG-IFNα + RBV + TVR	2016
Hemophilia + HCV infection GT 1-4	NCT01741545	Phase 3	51	180 μg PEG-IFNλ + RBV + DCV	2016
HCV infection GT 1-4	NCT01001754	Phase 2b	525	120/180/240 μg of PEG-IFNλ + RBV or 180 μg PEG-IFNα2a + RBV	2014
HCV infection GT 1-4	No NCT number provided	Phase 2a	55	80/120/180/240 μg PEG-IFNλ + RBV or 180 μg PEG-IFNα2a + RBV	2010
HCV infection GT 1	No NCT number provided	Phase 1b	56	1.5/3.0 μg/kg * PEG-IFNλ or 0.5–2.25 μg/kg * PEG-IFNλ + RBV or 1.5 μg/kg * + RBV	2010
Healthy volunteers	No NCT number provided	Phase 1a	24	0.5/1.5/5.0/7.5 μg/kg * PEG-IFNλ or placebo	2007

Data from http://www.clinicaltrials.gov accessed on 29 November 2024. GT: Genotype; HBV: Hepatitis B virus; HCV: Hepatitis C virus; HDV: Hepatitis D virus; PEG-IFN: Peginterferon; RBV: Ribavirin; LNF: Lonafarnib; RTV: Ritonavir; DCV: Daclatasvir; TVR: Telaprevir. * Dosing based on scaling of the 15 mg/kg NOEL (no observed effect level) determined in cynomolgus monkey model.

**Table 3:** Overview of clinical trials of pegylated-interferon lambda in patients infected with SARS-CoV-2.

Clinical Trial Number	Trial Phase	Number of Participants	Intervention	Publication Date
NCT04727424	Phase 3	1951	180 μg PEG-IFN-λ or placebo	2023
NCT04331899	Phase 2	120	180 μg PEG-IFN-λ or placebo	2021
NCT04354259	Phase 2	60	180 μg PEG-IFN-λ or placebo	2021

Data from http://www.clinicaltrials.gov, accessed on 29 November 2024. PEG-IFNλ: Peginterferon lambda.

## Data Availability

No new data were created or analyzed in this study. Data sharing is not applicable to this article.

## Abbreviations

The following abbreviations are used in this manuscript:

PEG-IFNα2a	Pegylated interferon alfa 2a
PEG-IFNλ	Pegylated interferon lambda
PEG-IFNα	Pegylated interferon alfa
SARS-CoV-2	Severe acute respiratory syndrome coronavirus 2
COVID-19	Coronavirus disease 2019
IFNLR1	Interferon lambda receptor 1
IL-10Rβ	Interleukin-10 receptor β chain
IFNAR	Receptor for type I Interferons
IFNLR	Interferon lambda receptor
ISGF3	Interferon stimulated gene factor 3
NRTIs	Nucleoside reverse transcriptase inhibitors
ISGs	Interferon stimulated genes
DAAs	Direct acting antivirals
HBeAg	Hepatitis B e antigen
IFNs	Interferons
IFNα	Interferon alfa
IFN-λ	Interferon lambda
IRF9	Interferon regulatory factor 9
STAT	Signal transducer and activator of transcription
JAK	Janus kinase
TYK	Tyrosine kinase
HBV	Hepatitis B virus
HCV	Hepatitis C virus
HDV	Hepatitis D virus
HEV	Hepatitis E virus
HIV	Human immunodeficiency virus
SVR	Sustained virologic response
RBV	Ribavirin
DCV	Daclatasvir
TVR	Telaprevir
CHD	Chronic hepatitis D
SLE	Systemic lupus erythematosus
LNF	Lonafarnib
RTV	Ritonavir
IL-10	Interleukin-10
GT	Genotype
